# Existential suffering in the day to day lives of those living with palliative care needs arising from chronic obstructive pulmonary disease (COPD): A systematic integrative literature review

**DOI:** 10.1177/02692163221074539

**Published:** 2022-02-17

**Authors:** Louise Elizabeth Bolton, Jane Seymour, Clare Gardiner

**Affiliations:** Division of Nursing & Midwifery, University of Sheffield, Sheffield, UK

**Keywords:** Chronic obstructive pulmonary disease, COPD, existentialism, palliative care, life meaning

## Abstract

**Background:**

The impact of living with palliative care needs arising from COPD disrupts an individual’s existential situation. However, no comprehensive synthesis of existing research has been published to determine the presentation and impact of existential suffering.

**Aim:**

To provide a synthesis of existing evidence on existential suffering for those living with palliative care needs arising from COPD.

**Design:**

This is an integrative review paper, undertaken using the methodological approach developed by Soares and reported in accordance with PRISMA guidelines. Data analysis was undertaking using an integrated convergent synthesis approach.

**Data sources:**

Nine electronic databases were searched from April 2019 to December 2019. A second search was undertaken in January 2021 to identify recently published papers meeting the inclusion and exclusion criteria. No date restrictions were imposed. Only papers published in the English Language were considered for inclusion. Empirical research papers employing qualitative and/or quantitative methodologies and systematic literature reviews were included. Articles were accepted for inclusion if they discussed any component of existential suffering when living with COPD and palliative care needs.

**Results:**

Thirty-five papers were included within this review comprising of seven systematic reviews, 10 quantitative studies and 18 qualitative studies. The following themes relating to existential suffering were found: Liminality, Lamented Life, Loss of Personal Liberty, Life meaning and Existential isolation. The absence of life meaning, and purpose was of most importance to participants.

**Conclusions:**

This review suggests existential suffering is present and of significant impact within the daily lives of those living with palliative care needs arising from COPD. The absence of life meaning has the most significant impact. Further research is required to understand the essential components of an intervention to address existential suffering for this patient group, to ensure holistic palliative care delivery.

**What is already known about the topic?**

- When living with palliative care needs arising from COPD, an individual’s existential situation is disrupted, often resulting in existential suffering.
- Much of global COPD palliative care policy is based upon the management of physical symptoms and not on existential problems, despite the negative impact existential suffering has upon quality of life.

**What this paper adds?**

- Patients living with palliative care needs arising from COPD experience existential suffering across many elements of their daily lives.
- Existential suffering is manifest in feelings of living in liminal space, a loss of personal liberty, isolation from social and family relationships alongside beliefs of life ceasing to exist.
- Identification of the limited literature available about the presence and absence of life meaning when living with palliative care needs arising from COPD and how these impact upon daily life.

**Implications for practice, theory or policy:**

- The need for a conceptual framework to guide patient care in COPD and address existential well being has been identified.
- This review highlights potential areas of concern in the daily lives of those living with palliative care needs arising from COPD, giving clinicians the knowledge to explore these when undertaking patient assessment.

## Introduction

There are an estimated 328 million people living with COPD globally,^1,2^ with predictions of it becoming the third biggest cause of death worldwide by 2030.^2^ Over 90% of COPD related deaths occur in low to middle income countries,^3^ with the condition receiving little interest and financial support when compared to other causes of international morbidity and mortality. This results in increasing challenges to the delivery of holistic and patient-centred palliative care.^4^

Whilst the physical symptoms of COPD can be severely debilitating, the impact of COPD extends beyond physical symptoms, disrupting an individual’s existential situation. The aim of this integrative review is to provide a synthesis of existing evidence on existential suffering for those living with palliative care needs arising from COPD, and explore its significance within, and impact upon, day to day life. Table 1 defines the key terms used within this paper. Patients with COPD survive many exacerbations of their disease, with deteriorating quality of life and symptoms that can be more troublesome than those experienced by people with lung cancer.^5–7^ Timely and consistent transitions to palliative care remain absent,^8^ and although models of palliative care for patients with COPD are well-evidenced, they are largely focussed upon the relief of physical symptoms.^9–11^

**Table 1.** Key terms used within integrative review.

Key term	Definition
Palliative care needs	The individualised needs of a person including assessment and management of physical symptoms, psychological and social needs.^12^
Existential situation	An individual’s human existence, freedom, and choice to allow them to make decisions as a conscious being.^13^
Existential suffering	The absence of life meaning, purpose hope and social connectedness, posing threat to one’s personal identity.^14^

The unpredictable disease trajectory of COPD causes delays in commencing palliative care. Limited understanding of the COPD disease trajectory among healthcare professionals,^15^ results in a reactive approach to COPD exacerbation management,^6,16^ rather than the delivery of active treatment and palliative care concurrently.^7^

### Existentialism

Conceptually, existentialism explores the human existence, and focuses upon lived experiences through thoughts, feelings and actions. Existentialism describes how our existence constructs meaning about what is important and has purpose within our daily lives.^13^ Existential disruption occurs when the importance and purpose within life becomes unidentifiable, resulting in existential suffering. In the context of its emotional effects, existential suffering leads to feelings of fear, guilt and a lack of self-worth,^17–19^ exacerbated by the associated meanings attached to these symptoms.^19^ If unaddressed, disruption to an individual’s existential situation results in them feeling incomplete and lacking a sense of peace.^19,20^ Existential suffering often remains undisclosed by individuals, in an attempt to maintain personal integrity and some state of normality,^19,21^ yet the deep-rooted effect of this experience manifests as an inability to live a fulfilling life. Existing literature demonstrates that existential suffering is one reason why patients may wish to end their lives.^22,23^ Understandings of individuals’ existential situations within COPD are limited in the research literature and are rarely addressed within clinical practice,^24^ yet existential suffering has been linked to poor health-related quality of life for those living with other chronic conditions.^25,26^

### Existential suffering in COPD

When those living with COPD are nearing the end of life, associated physical and mental symptoms preoccupy them.^27^ Some individuals, despite the daily overwhelming symptom burden, remain unwilling to discuss death and dying and redirect their hopes and desires elsewhere in search of stability and normality.^27–30^ This coping mechanism, often grown from feelings of guilt and shame,^31^ inhibits future care planning.^27^

The need for interventions designed to address elements of existential suffering in COPD is evident. People with other chronic illnesses with similar symptoms to COPD have also been found to experience existential suffering. Interventions focussed upon alleviating elements of existential suffering have been reported as effective in patients living with cancer, heart failure and chronic kidney disease, with participants reporting a greater sense of well-being and inner peace.^25,26,32^

Within the wider palliative care literature, existential suffering remains under researched with an absence of evidence-based tools and interventions to assess, plan care and facilitate the alleviation of symptoms. Existential suffering is a complex and individualised concept^14^ and inconsistent definitions as well as limitations of the evidence base present difficulties for healthcare providers in responding to this patient need.^14,23^

Despite the exploration of elements contributing to, and impact of existential suffering in day-to-day life in COPD, no synthesis of the literature exists. Understanding the degree of impact of existential suffering for those living with palliative care needs arising from COPD will allow healthcare professionals to explore the necessary components of assessment, care planning and intervention content to enhance palliative care delivery. The aim of this integrative review is to provide a synthesis of existing evidence on existential suffering for those living with palliative care needs arising from COPD, and explore its significance within, and impact upon, day to day life.

## Method

### Design

This is an integrative review paper, following methodological approaches developed by Soares et al.,^33^ to allow for the merging of findings from differing data types, to develop an inclusive understanding of a particular topic.^34^ Using this interpretivist approach allowed the development of an understanding of how the phenomenon of existential suffering is present within the daily lives of those living with COPD.^35^ Insights into the deep meanings of individuals experiences were able, with consideration given to differing cultures, circumstances and social realities. The review was reported in accordance with PRISMA guidelines.

### Search strategy

A pre-defined keyword search of the following nine electronic databases was performed in March to December 2019: Web of Science Core Collection, CINAHL, Cochrane Systematic Review Database, EThos – Thesis database, PsychINFO, SCOPUS, PROSPERO, ASSIA and Google Scholar. A second search was undertaking in January 2021 to identify any further published papers meeting the study inclusion criteria. No new papers were identified. The search terms used are displayed within Table 2.

**Table 2.** Pre-defined integrative review search terms.

COPD search terms	Existential suffering search terms
‘Chronic obstructive pulmonary disease’	‘Existential suffering’
COPD	‘Existential distress’
Emphysema	Meaninglessness
‘Respiratory Disease’	Life meaning
‘Respiratory conditions’	Hopelessness
‘COPD management’	‘Absence of hope’
‘COPD treatment’	Purposefulness
‘COPD interventions’	Existential
‘COPD exacerbation’	Existentialism
‘Pulmonary disease’	Anxiety
‘Chronic obstructive airways disease’	‘Anxiety management’
COAD	Depression
‘Chronic bronchitis’	Depressive
	‘Low mood’
	Resilience
	Emotional
	Emotion
	Suffering
	Loneliness
	Meaning
	‘Meaning making’
	Worthlessness
	‘Existential therapy’
	‘Existential anxiety’
	‘Existential crisis’
	‘Existential counselling’

**Figure 1.**
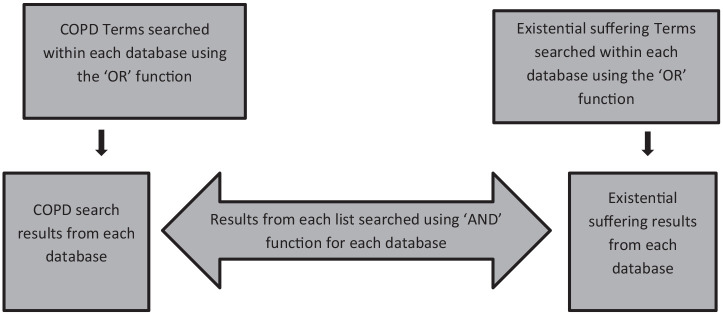
Database search process.

The COPD and existential suffering search terms were searched initially as individual lists using the ‘OR’ function to focus the search by connecting similar concepts, as demonstrated in Figure 1. The ‘AND’ function was then used to further focus the search to produce results related to existential suffering in COPD.

A total of 68,427 citations were identified for title and abstract screening. The large number of initial search results came due to the use of broad terms such as ‘respiratory disease’, ‘pulmonary disease’ and ‘respiratory conditions’ which identified papers not related to COPD but other respiratory conditions. These terms were necessary for inclusion as they did produce some relevant results that would have otherwise been missed. Weekly database searches were undertaken until December 2019 and a final search in January 2021. No date restrictions were placed upon the searches.

### Inclusion and exclusion criteria

The study inclusion and exclusion criteria are provided in Table 3. These criteria were developed prior to any data searches by the review team. The inclusion criteria use descriptive indicators to best reflect participants within included studies whom have palliative care needs arising from COPD.

**Table 3.** Integrative review inclusion and exclusion criteria.

Inclusion criteria	Exclusion criteria
Published in the English Language	
Participant population of adults aged over 18 years	
Focussed upon the exploration of components of existential suffering when living with COPD and associated palliative care needs	Studies relating to the exclusive concept of spirituality or religion without the inclusion of any of the previously explained elements of existential suffering.
	Studies about reducing anxiety and depression in those living with advanced COPD and associated palliative care needs where the outcome measures are not related to components of existential suffering
Participants identified by the authors as having advanced COPD by use of one or more of the following descriptive indicators: Advanced COPD Severe COPD Very severe COPD End Stage COPD Palliative COPD Palliative Care needs End of life needs	
Empirical studies – Qualitative, Quantitative and mixed-methods, Systematic Literature Reviews, Single case studies, Intervention studies, Randomised controlled trials, PhD Theses	Grey Literature

### Data search

**Figure 2.**
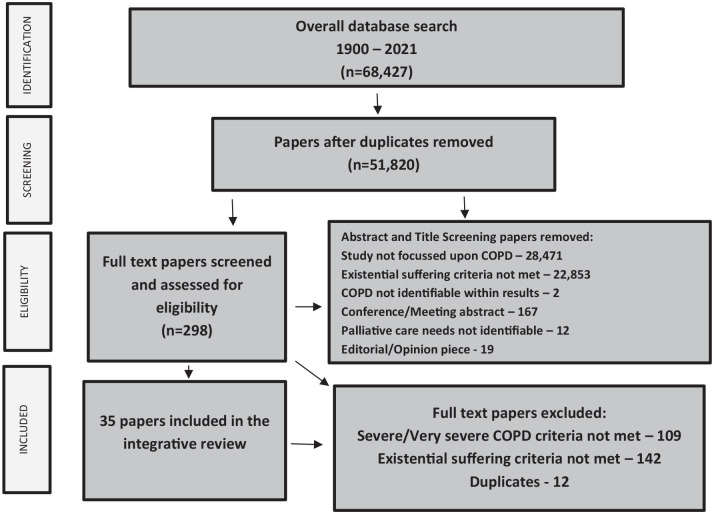
PRISMA flowchart.

The study selection process is displayed within Figure 2, using the Preferred Reporting Items for Systematic Reviews and Meta Analyses (PRISMA) Flowchart. Following title, abstract and full-text screening by the lead author, 35 papers were included within this review. Discrepancies upon the inclusion of a paper were discussed within the review team and a decision made upon inclusion or exclusion. For papers meeting the study inclusion criteria whereby only some of the included participants had advanced COPD, a practical approach was applied. Papers whereby individual participant data/responses could be identified within the findings, then those responses were included within the review. In the case whereby individual responses could not be identified, those papers were excluded from the review.

### Data extraction and analysis

Data from each study were extracted to identify study characteristics and establish study quality. A data extraction tool, developed for this review, allowed organisation of study information including study aims, study design, sample selection, data collection, data analysis and findings.

Data analysis was undertaken using integrated convergent synthesis to develop themes relevant to the review aims. This involved the identification of themes from the data presented in qualitative and quantitative studies separately, followed by identification of meta-themes across all study types. The approach allowed for little consideration to be given to the methodological approaches of each included paper, allowing for integration and synthesis of study findings based upon their focus all being upon the same concept.^36^ The qualitative data analysis software QUIRKOS was used to store and organise the data into themes. The initial analysis was completed by the lead author. The second and third authors reviewed the themes for accuracy and representation of the initial data prior to confirmation.

### Quality assessment

Data evaluation and quality assessment within an integrative review is a complex process lacking clear guidance.^37,38^ Therefore, quality assessment tools specific to each study type were adopted, to evaluate the specific methodological features of each included study,^36,37^ The following quality assessment tools were used; Critical Skills Appraisal Programme Checklist for Qualitative Research,^39^ Checklist for Quasi-Experimental Studies (The Joanna Briggs Institute)^40^ and for mixed method-studies both tools were used for relevant study components. The quality assessment determined the methodological rigour of each included study alongside the risk of bias. Studies of poor methodological rigour carried less weight within the review analysis but were not excluded.

## Findings

A total of 35 papers comprising seven systematic reviews,^41–46^ 10 quantitative studies^47–56^ and 18 qualitative studies^28–31,57–70^ were included. Study characteristics and a summary are provided in Table 4. 1453 participants were included within the 35 studies, of whom 1090 had severe or very severe COPD. Data presented from study participants at an earlier stage in COPD disease progression or in the absence of palliative care needs were excluded from this review. All included studies were of high methodological rigour and contained minimal risk of bias.

**Table 4.** Included study characteristics (*n* = 35).

Author Country	Journal	Participant characteristics	Study design	Recruitment setting and sample size	Methods	Analysis	Findings
1. Brien et al.^65^ *United Kingdom*	Primary Care Respiratory Medicine	9 participants had a very high score on health related QOL impact of COPD. 14 participants classified as having either severe or very severe COPD	Purposive sample from primary and secondary care sites – maximum variation sample across disease severity.	34 Participants Primary and Secondary Care settings	Semi-structured interviews	Thematic analysis	Coping strategies commonly used are medication, pacing with activities of living and distraction. Challenges to coping are psychological and co-morbidities
2. Cooney et al.^66^ *United Kingdom*	Journal of Clinical Nursing	Participants from within PRINCE RCT (2012) – 31.5% of intervention group (interviewed in this study) had severe COPD	Grounded Theory Purposive sample leading to theoretical sample from primary care settings.	26 participants 15 Male/11 Female Primary Care setting	Semi-structured Interviews	Constant comparative technique	The theory of-co existing is prevalent within COPD, particularly when managing breathlessness
3. Cruz et al.^41^ *Portugal*	Health and Social Care in the Community	Included studies had participants with severe/advanced/terminal COPD	Integrative Review	18 included studies	Liberati et al. (2009) principles of a systematic search were followed	Thematic Synthesis	Caring for someone with COPD is a stressful experience, carers perceived a loss of identity and personal freedom.
4. Disler et al.^42^ *Australia*	Journal of Pain and Symptom Management	Review focus upon individuals with advanced COPD	Qualitative Systematic Review	22 included studies.	PRISMA guidelines followed	Meta-synthesis of Qualitative data. Thematic synthesis – descriptive and analytical themes	Themes identified for use when caring for those with COPD included a better understanding of the condition and the unrelenting psychological impact.
5. Dunger et al.^67^ *Germany*	Palliative and Supportive Care	Sample constructed of participants with Severe or Very severe COPD. Focus upon symptoms at end of life	Qualitative longitudinal study	10 participants with COPD 4 Male/6 Female. Primary and secondary care including palliative care units	Topic-guided in-depth interviews	Framework analysis	The impact of breathlessness causes disruption to how life is lived. This disruption causes feelings of despair and hopelessness.
6. Ek and Ternestedt^31^ *Sweden*	Journal of Advanced Nursing	All participants with COPD were in the palliative phase of disease progression	Giorgi’s phenomenological method	8 participant’s Secondary care	Semi-structured interviews	Phenomenological-hermeneutical analysis	Living with COPD in a couple resulted in living with uncertainty, a changed intimate relationship and developing new ways of living together
7. Ek et al.^68^	Journal of Palliative Care	Considered to be in the final phase of COPD disease progression	Phenomenological-hermeneutical methodology	4 couples/8 participants Primary care	Repeated interviews over an eight-month period	Dialectical movement analysis	An awareness of the importance of personal values facilitates daily structure
8. Elofsson and Ohlén^28^ *Sweden*	Palliative Medicine	Advanced COPD	Phenomenological-hermeneutical methodology	6 participant’s Secondary care	Narrative dialogues	Phenomenological analysis	Participants had feelings of resignation and sadness. They had little interest in hobbies and found living with COPD to be a hard life. Socialising was important to give the individual a sense of identity.
9. Gabriel et al.^69^ *Portugal*	Psychology & Health	60% of participants had severe to very severe COPD	Exploratory Qualitative Study	20 patients – 16 male/14 female 20 family member’s – 12 spouses, 8 adult children Primary and Secondary care.	Open-ended question interviews	Descriptive statistical thematic analysis	Coping strategies used to handle the difficulties of living with COPD included socialisation, help from professional networks and seeking relevant information about COPD.
10. Gale and Sultan^70^ *United Kingdom*	Health and Place 21	6 participants had severe/very severe COPD	Intervention study	5 male/2 female Community setting	Situated interviewing	Thematic analysis	The telehealth intervention gave participants peace of mind through contact with healthcare professionals and through increasing their own self-confidence.
11. Gardener et al.^43^ *United Kingdom*	International Journal of COPD	Within included studies, participants all symptomatic of breathlessness – palliative need	Qualitative systematic review	31 included papers	PRISMA guidelines followed	Thematic analysis mapped to palliative and end of life care policy.	Identified support needs of patients included understanding COPD, managing feelings and worries, families and close relationships and social and recreational life
12. Gardener et al.^57^ *United Kingdom*	Palliative Medicine	All patient participants had advanced COPD	Two-stage qualitative study	20 patients	Focus groups	Content analysis	Development, review, and refinement of patient support needs tool to enable delivery of person-centred care
13. Harb et al.^58^ *Australia*	International Journal of COPD	Severe COPD	Qualitative study design	26 participants	Semi-structured interviews	Framework analysis – using establishes treatment burden framework	COPD has a substantial treatment burden. Patients are less likely to accept medical treatment if they perceive the benefit to be insufficient
14. Hayle et al.^59^ *United Kingdom*	Palliative Medicine	Participants receiving palliative care	Phenomenological-hermeneutical methodology	8 participants Community or Hospice setting	Semi-structured interviews	Hermeneutic phenomenological approach	Specialist palliative care was perceived to have a positive impact upon psychological symptoms. Opportunities to improve palliative care for this group remain
15. Lee et al.^60^ *Canada*	Physiotherapy Theory and Practice	Participants had severe or very severe COPD and pain	Phenomenological study	8 participants Community setting	Semi-structured interviews	Interpretive Phenomenological Analysis	COPD participants found difficulty in explaining pain resulting in feelings of frustration and loss of self-worth
16. Lindqvist and Hallberg^29^ *Sweden*	Journal of Health Psychology	Participants had severe COPD	Grounded Theory	23 participant’s Secondary care	Semi-structured interviews	Grounded theory	Suffering from COPD resulted in feelings of guilt because of self-infliction. Linked to management strategies including making sense of existence, adjustment to bodily restrictions, surrendering to fate.
17. Lovell et al.^44^ *United Kingdom*	Journal of Pain and Symptom Management	Participants within included studies had severe/very severe COPD	Qualitative systematic review	38 included studies	PRISMA guidelines followed	Thematic synthesis	The importance of social participation and activities is of importance to individuals with COPD
18. Marx et al.^30^ *Germany*	BMJ Open	All participants had advanced COPD	Qualitative longitudinal study	Community setting 17 participants	Narrative semi-structured interviews	Grounded theory	Patients with COPD have difficulties accepting their life situation and feel at mercy of the disease.
19. May et al.^45^ *United Kingdom*	BMJ Open	Papers included participants with severe and very severe COPD	Qualitative systematic review	53 included papers	Qualitative content analysis	Thematic synthesis	People living with COPD have significant pathophysiological deterioration. COPD disrupts social networks and gives associated feelings of dependence and vulnerability.
20. Olsman et al.^61^ *Netherlands*	Palliative and Supportive Care	Participants with COPD defined as severe	Qualitative longitudinal method	Community and Hospice setting 29 participants (10 with COPD)	Semi-structured interviews	Thematic analysis	COPD presents feelings of hope for the future, hopelessness, and despair.
21. Russell et al.^46^ *United Kingdom*	Primary Care Respiratory Medicine	Papers included participants with severe and very severe COPD	Qualitative systematic review	33 studies included in review	Qualitative content analysis	Thematic analysis	Over time, COPD can consume existence and reduce motivation. Family support may prove vital yet trigger feelings of being a burden
22. Sheridan et al.^62^ *New Zealand*	Primary Care Respiratory Journal	Participants had from moderate to very severe COPD	Qualitative methodology	29 participants Community setting	Semi-structured interviews	Thematic analysis	All participants expressed feelings of helplessness in managing their condition.
23. Strang et al.^63^ *Sweden*	Palliative and Supportive Care	Papers included participants with severe and very severe COPD	Qualitative methodology	31 participants Community and clinic settings	In-depth interviews	Thematic content analysis	Three themes identified contributing to anxiety associated with COPD – death anxiety, life anxiety and counterweights to anxiety.
24. Stridsman et al.^64^ *Sweden*	Primary Health Care Research & Development	Participants had severe and very severe COPD	Qualitative methodology	10 participants	Semi-structured interviews	Latent qualitative content analysis	Participants adjusted to new limitations through acceptance and undertaking new activities
25. Chochinov et al.^47^ *Canada*	PLoS ONE	Participants with very severe COPD	Prospective multi-site approach	100 participants with very severe COPD Outpatient departments, care homes, inpatient care settings	Questionnaires including: Structured Interview of Symptoms and Concerns, Herth Hope Index, Spiritual Survey, Patient Dignity Inventory and	Descriptive statistics using Patient Dignity Inventory.	Participants with COPD face a loss of personal dignity. Patterns of existential distress identified.
26. Doyle et al.^48^ *Australia*	British Journal of Health Psychology	Participants had severe COPD	Pragmatic Randomised Controlled Trial	95 Participants Community setting	Intervention – CBT or Befriending service	Intention to treat analysis	CBT Therapy reduced depression symptoms but not anxiety.
27. Harrison et al.^49^ *Canada*	Chronic Respiratory Disease	9 out of the 15 participants had severe or very-severe COPD	Two-stage mixed methods study	15 participants Community setting	Mixed method study. Semi-structured interviews and Questionnaires	Inductive thematic analysis Pearson correlations	Self-conscious emotions were related to elevated anxiety and depression.
28. Ivziku et al.^50^ *Italy*	Quality of Life Research	49 of the participants had severe or very severe COPD	Cross-sectional descriptive design	80 Participants Outpatient setting	Patient health questionnaire Generalised Anxiety Disorder questionnaire 12-Item Short form health survey	Descriptive statistics Pearson’s product-moment correlation coefficients	Caregivers psychological distress influences patient’s physical quality of life.
29. Keil et al.^51^ *Germany*	Chronic Respiratory Disease	406 of the participants had severe or very severe COPD	Online study	Community setting 531 participants	Online survey utilising COPD disability index, Hospital Anxiety and Depression Scale, Antonovsky’s sense of coherence scale, 13-item Resilience Scale	Multiple linear regression analyses	Sense of coherence and resilience hold potential to assist adjustment to living with COPD.
30. Low et al.^52^ *Canada*	Western Journal of Nursing Research	38 of the participants had severe to very severe COPD	Cross-sectional survey study	87 Participants Community Setting	Questionnaires: St. George’s Respiratory Questionnaire, Attitudes to Aging Questionnaire.	Multiple analysis of variance	Participants downplayed their symptoms of COPD and psychosocial impact.
31. Reijnders et al.^53^ *Germany*	Journal of Chronic Obstructive Pulmonary Disease	41 participants had severe or very severe COPD	Outcome measurement study	Inpatient setting 104 participants	Face to face questionnaire completion – 6-minute walk test, De Jong Gierveld Loneliness Scale, The patient health questionnaire, Health-related quality of life questionnaire	Hierarchical linear regression	Loneliness identified in COPD and impacts upon ability to undertake pulmonary rehabilitation. Loneliness associated with poor exercise function.
32. Stenzel et al.^54^ *Germany*	Psychology & Health	101 participants had severe or very severe COPD	Longitudinal physical examination and questionnaire	131 participants Inpatient setting	Self-report questionnaires	Regression and mediation analysis.	End of life care should not only be based upon physical illness symptoms but also upon psychological distress and disease-specific anxieties.
33. Vaske et al.^71^ *Germany*	Journal of Health Psychology	342 participants had severe or very severe COPD	Online survey design	444 participants Secondary Care	Online Questionnaires: Illness Perceptions Questionnaire/Essener Coping Questionnaire/HR-QOL Short Form	Hierarchical regression and moderation analysis	To prevent reduced HR-QOL in COPD, treatment needs to include promotion of coping with the disease and functional illness perceptions.
34. Vitacca et al.^56^ *Italy*	Journal of Palliative Medicine	All participants had less than a 1-year life expectancy	Intervention study	Inpatient and outpatient settings 10 participants	Self-report questionnaires	Data expressed as an absolute number of percentages.	Bad days of life, negative emotions and perception of disease deterioration were topics discussed by patients. Telehealth is accepted by patients.
35. Rosa et al.^72^ *Italy*	Nursing Open	Participants within included papers had severe or very severe COPD	Mixed methods Systematic Review	20 papers reviewed	Rapid evidence assessment	Thematic synthesis	Resilience is a useful concept when understanding family caregiving within COPD.

**Figure 3.**
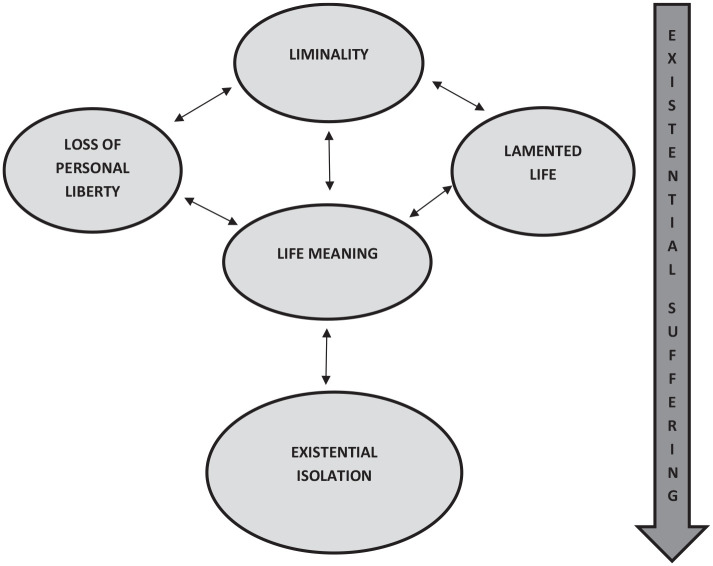
Conceptual diagram of existential suffering for those living with palliative care needs arising from COPD.

The studies explored a range of issues relating to COPD, all directly or indirectly related to existential suffering. Five themes were identified (demonstrated within Figure 3): Liminality, Loss of Personal Liberty, Lamented Life, Life Meaning and Existential Isolation. The influence of each theme is multi-directional, with each element of existential suffering contributing to the development of another.

### Liminality

For those living with advanced COPD and associated palliative care needs, the feeling of living in a liminal space was identified from 15 studies.^28–31,42,49,58,60,65–70,72^ This involved a sense of uncertainty and a continuous effort to process the reality of their new lives through reflection in the past and future. Participants no longer perceived themselves to be the same individual yet were unable emotionally to determine who they now were as a person. Feelings of loss and grief for their former selves and lives contributed to feelings of uncertainty around their future selves, complicated by the anticipation of how COPD disease progression will present.

Negative self-perception exacerbated the feeling of living within a liminal space.^28–31,42,49,60,65,68–70^ Some participants experienced embarrassment about their new identity. Feelings of worthlessness and being unable to successfully undertake daily life tasks added to this.^30,31,42^ This attack on individuals’ personal identity created strong emotions around difficulties with social acceptability.

Denial of having COPD was evident across three of the included studies.^29,42,67^ This facilitated feelings of living in a liminal space, hindering adaptation to new life circumstances.^29,30^ Participants who had to some degree accepted the impact of the disease, felt forced to do so because of the overwhelming symptom burden.

### Lamented life

The theme of a lamented life captured thoughts of emptiness, hopelessness, worthlessness and desolation.^28,29,31,42,45–47,49,50,53,54,56–58,60–63,65,67–70^ The experience of living with palliative care needs arising from COPD presented as a continuous stream of losses. Living within a liminal state influenced the experience of hopelessness and emptiness as a result of being unable to identify how life will be in the future. Participants felt an absence of hope within their lives, perceiving a lack of future and immense sadness at their current situation.^28,29,31,42,50,56,58,60–62,65,67^ Thoughts of grief about the loss of former life manifested into a lack of life purpose,^31,42,73^ stimulated by the lack of ability to plan a fulfilling future life.^29,31,42,60,61,67^

Feelings of diminishing usefulness within daily life were underpinned by a loss of ability to accomplish tasks, resulting in feelings of worthlessness and low self-esteem.^31,42,60,69^ One participant perceived their life to be so worthless, that they voiced: ‘If you were a dog, the RSPCA would have you up for keeping me alive’.^42^ Observing others undertaking tasks that participants could no longer complete led to feelings of sadness and worthlessness.^31,69^

Participants reported the absence of a meaningful life existence, and described life being empty with little reward, which led to feelings of desolation.^28,31,42^ Some believed that their friends no longer wanted to socialise with them due to their COPD. Participants welcomed input from healthcare professionals to facilitate interventions to address these feelings.^31,68^

### Loss of personal liberty

Participants reported significant frustration that they could no longer pursue pleasurable life activities and hobbies, alongside their previous activities of daily living.^28,31,42,65,66^ This loss caused one participant to lament the loss of their former life: *‘there’s a lot you can’t do. . .a great deal, and much you have to give up. . .’*,^31^ leading to challenging thoughts about life meaning and purpose.

Feelings of life being ‘just an existence’ were evident, with one participant describing their meaningless day: *‘So essentially my day consists of nothing more than resting and reading a little; no, this isn’t living, it’s just existing. . ...I just am’*. ^31^ The impact of existing in a life without meaning caused some participants to wish they were no longer living,^31^ with one participant discussing feelings of suicide: ‘and then you feel awful and at that point you just want to get away from everything, even commit suicide, you feel like there’s just no point’.^31^ Restrictions due to the lack of ability to leave home were found to have the greatest impact.^52,69^

### Life meaning

Some elements of participants’ lives facilitated a sense of meaning and purpose, allowing for distraction from illness and current health state.^28,29,31,42,43,48,51,53,56,59,62–65,67^ Attitudes to their illness influenced how participants adapted to their limited life. Some were still able to identify contributions to society giving them a life purpose: *‘I have so much to give – I’m great with teenagers, I’m a good grandmother, I’m a good wife, that’s what I believe’*.^31^

Maintaining or discovering hobbies within their physical limitations provided participants with a focus for their time, allowing them to feel a sense of achievement.^28,31,65^ Assisted trips outside of the home, being able to feel connected to society by looking out of the window, alongside caring for pets enhanced feelings of pleasure and meaning.^31,63^

Participants voiced strategies to distract from symptoms and feelings of anxiety, including maintaining a good sense of humour, the application of positive thinking and abstaining from worrying through ‘taking life as it comes’.^42,65^

The value and impact of interactions with healthcare professionals and access to supportive interventions was identified as a sub-theme influencing life meaning.^43,48,53,56,59,64,70^ An overwhelming sense of social connection through attendance at intervention based sessions gave participants increased confidence and peace of mind when living with such unrelenting symptom burden, working towards overcoming social isolation.^48,56,57,70^

### Existential isolation

Feelings of being alone in one’s own existence developed into feelings of being misunderstood, resulting in loneliness and frustration. The impact upon intimate relationships forced the reshaping of relationship dynamics to facilitate coping.^68,69^ Intimate and sexual relations were often lost, contributing to existential isolation within personal relationships.^41,42,44,66,68^

The greatest challenge of maintaining social relationships was the limited opportunities to meet with friends and socialise within their local community, due to physical limitations imposed by illness.^28,42–44,51,53,57,58,62,66,69^ Physical symptoms such as a chronic cough and excessive sputum production reduced the desire to socialise, with some believing friends declined interactions as a result.

## Discussion

This integrative review provides a synthesis of international evidence about the presence of existential suffering for those living with palliative care needs arising from COPD. The evidence suggests existential suffering significantly impacts upon the daily lives of those living with COPD. Living in a state of liminality, originating from patient’s loss of identity and life role results in feelings of loneliness, worthlessness and desolation. The absence of life meaning and purpose because of physical and emotional restrictions compound existential isolation.

Liminality is conceptualised as a lived experience whereby individuals are in an ambiguous state of being ‘neither one thing or another’.^74^ Literature on late-stage cancer identifies a state of liminality occurring when living and dying occur concurrently,^75^ a notion transferable to the COPD disease trajectory. Living within a liminal space gives rise to an ambiguous life state and social separation,^76^ influencing existential suffering through the generation of loneliness, hopelessness and meaninglessness in daily life. McKechnie et al.^76^ argues that on arriving at a liminal state, through suffering from an incurable illness, the individual is unable to return to a pre-liminal state. This integrative review aligns with the wider literature, identifying that existential suffering and associated liminality can be addressed through specifically designed interventions.^77^ Limitations of available literature within the COPD population highlight the need for further research in this field. Findings from quality of life intervention studies in COPD demonstrate relief from some symptoms of existential suffering.^78–80^ In one of these,^79^ 23 participants received a healthcare intervention aimed to improve quality of life in patients with COPD. Participants reported enhanced social and emotional support, feelings of success and a positive improvement in mood which contributed to their existential wellbeing. These findings are echoed within an intervention study focussed on managing anxiety.^78^ The intervention facilitated increased feelings of control alongside opening discussions on end-of-life concerns. Despite this, little is known about the most impactful intervention elements and style of delivery to relieve existential suffering.

Our review found the loss of an individual’s personal liberty when living with palliative care needs arising from COPD leads to challenges to life purpose, particularly when they are no longer able to undertake pleasurable activities. Furthermore, having the motivation to undertake hobbies, activities or plans of treatment is inhibited through perceptions of ‘just existing’. These findings align with the wider literature about the lack of motivation to complete treatment programmes, in people with COPD.^81–83^ A study evaluating reasons for non-compliance of pulmonary rehabilitation programmes found 49% of the 126 participants cited a lack of motivation as their main reason.^81^ Interventions have been developed and evaluated to address non-compliance with treatment programmes in COPD, some of which aim to address elements of existential suffering. A motivational intervention including a component to provide participants with a positive life perspective demonstrated an increase in motivation when self-managing COPD.^84^ This highlights the need for further research exploring improvements in treatment, care and medication compliance through addressing existential suffering in COPD.

Our review demonstrates that the manifestation of meaninglessness and its physical and psychological impact has been little explored in COPD. The few studies that have explored this are based on small numbers of participants, hindering generalisability of results.^28–30^ Explorations of meaninglessness in cancer and other long-term conditions with similar symptom burden do exist within the wider literature. For those who have accepted they are approaching death, the significance of meaninglessness becomes more prevalent.^85^ Rediscovering life meaning within such a significant life phase allows individuals to transfer feelings of hopelessness and guilt into an engaged and purposeful response to daily life,^85^ yet how this knowledge translates into clinical practice is unclear. A study of 456 health care professionals’ perceptions of the effectiveness, necessary approach and content of an intervention to address existential suffering in terminally-ill cancer patients identified that a meaning-centred approach, comprising of life-review interviews, cognitive restructuring and exploring life values was regarded as the most effective approach.^77^ Our integrative review supports these findings. Included studies^56,59^ have facilitated an exploration of life values, with participants voicing feelings of enhanced worth and a sense of control over life.

The need for a conceptual framework to guide patient care in COPD and address existential well-being is evident from this review. The themes identified have added to the existing evidence base identifying possible components of a framework, but further research is required to develop and evaluate interventions. Further work is required to understand the necessary intervention components when addressing existential suffering in COPD. Furthermore, research is required to determine the most acceptable and effective intervention delivery method.

### Implications for clinical practice

This review provides clinicians with evidence about the significant impact of existential suffering on those with advanced COPD. In turn, this identifies the need to explore effective solutions. Clinicians are advised to consider the presence of existential suffering in the daily lives of those living with palliative care needs arising from COPD when planning and evaluating care. This paper gives suggestions upon how existential suffering may present, giving opportunities for further discussion to ensure a holistic approach to palliative care is adopted.

Social prescribing may be a way to begin addressing the impact of existential suffering, through referral to psychological support services or specialist respiratory support groups where appropriate. Utilising local befriender services may also be of benefit to this patient group.

Significant gaps in knowledge on existential suffering in palliative care for those living with COPD have been identified within this review. Liminality requires further exploration as a significant concept impacting upon the daily lives of those living with palliative care needs arising from COPD. This review highlights the importance of individuals feeling they live between two lives, resulting in feelings of meaninglessness and hopelessness impacting on the desire to engage in treatment and symptom control.

## Strengths and limitations of the review

Limitations of an integrative review methodology are acknowledged due to the complex process of integrating empirical and theoretical evidence from differing methodologies.^38^ As such, reduced rigour and the presence of bias may become present and has been of consideration throughout this review. Whilst an extensive database search was undertaken, searches were limited to the English and American English languages and a search of grey literature was not undertaken. This may have resulted in relevant papers being omitted. This review is necessarily limited by the data presented by the original authors of each included paper. Database searches were undertaken from April 2019 to January 2021. Any relevant studies published after this date have not been included yet may be of relevance. This review was undertaken prior to the Covid-19 pandemic and it is recognised that the daily lives of those living with COPD have experienced considerable effects. It is speculated that existential suffering has worsened throughout this time.

## Conclusion

Existential suffering plays a significant role in the daily lives of those living with advanced COPD and associated palliative care needs. Its presentation is an experience unique to everyone, with different elements being of significance to each person. The importance of experiencing meaning in life is the most significant element of existential suffering for those living with COPD. Upon rediscovery of life meaning, and diminishing feelings of worthlessness and hopelessness, a sense of inner peace may be established. For those living with such a relentless symptom burden, a hopeful existential situation is desirable but difficult to achieve. Further research is needed to explore the impact of meaninglessness, both physically and psychologically within the daily lives of those living with palliative care needs arising from COPD, and to develop interventions that support meaningfulness in COPD.

## Supplemental Material

sj-pdf-1-pmj-10.1177_02692163221074539 – Supplemental material for Existential suffering in the day to day lives of those living with palliative care needs arising from chronic obstructive pulmonary disease (COPD): A systematic integrative literature review Click here for additional data file. Supplemental material, sj-pdf-1-pmj-10.1177_02692163221074539 for Existential suffering in the day to day lives of those living with palliative care needs arising from chronic obstructive pulmonary disease (COPD): A systematic integrative literature review by Louise Elizabeth Bolton, Jane Seymour and Clare Gardiner in Palliative Medicine
