# Supplementary material for: Existential suffering in the day to day lives of those living with palliative care needs arising from chronic obstructive pulmonary disease (COPD): A systematic integrative literature review
Source: Palliat Med. 2022 Feb 17;36(4):567–80. doi: 10.1177/02692163221074539 (PMC9006392; doi:10.1177/02692163221074539)
Supplement: sj-pdf-1-pmj-10.1177_02692163221074539 – Supplemental material for Existential suffering in the day to day lives of those living with palliative care needs arising from chronic obstructive pulmonary disease (COPD): A systematic integrative literature review [file sj-pdf-1-pmj-10.1177_02692163221074539.pdf]

Search Strategy – Web of Science Core Collection

- Each term searched individually:

COPD Search Terms	Existential Suffering Search Terms
'Chronic Obstructive Pulmonary Disease'	'Existential Suffering'
COPD	'Existential distress'
Emphysema	Meaninglessness
'Respiratory Disease'	'Life meaning'
'Respiratory Conditions'	Hopelessness
'COPD Management'	'Absence of hope'
'COPD Treatment'	Purposefulness
'COPD Interventions'	Existential
'COPD Exacerbation'	Existentialism
'Pulmonary Disease'	Anxiety
'Chronic Obstructive Airways Disease'	'Anxiety management'
COAD	Depression
'Chronic Bronchitis'	Depressive
	'Low mood'
	Resilience
	Emotional
	Emotion
	Suffering
	Loneliness
	Meaning
	'Meaning making'
	Worthlessness
	'Existential therapy'
	'Existential anxiety'
	'Existential crisis'
	'Existential counselling'

- Results from COPD Search Terms combined using 'OR' function – Creating 'combined COPD search'
- Results from existential suffering search terms combined using 'OR' – Creating 'combined existential suffering search'.
- 'Combined COPD search' and 'combined existential suffering search' merged using 'AND' function
